# Effects of a High-Grain Diet With a Buffering Agent on Milk Protein Synthesis in Lactating Goats

**DOI:** 10.3389/fvets.2021.696703

**Published:** 2021-07-06

**Authors:** Meilin He, Xintian Nie, Huanhuan Wang, Shuping Yan, Yuanshu Zhang

**Affiliations:** ^1^The Key Laboratory of Animal Physiology and Biochemistry, Ministry of Agriculture, Nanjing Agricultural University, Nanjing, China; ^2^College of Engineering, Nanjing Agricultural University, Nanjing, China

**Keywords:** buffering agent, lactating dairy goats, HPLC, comparative proteomics, amino acids, milk protein

## Abstract

Chinese dairy industries have developed rapidly, providing consumers with high-quality sources of nutrition. However, many problems have also appeared during the development process, especially the low quality of milk. To improve milk quality, a large amount of concentrated feed is usually added to the diet within a certain period of time, which increases the milk production to a certain extent. However, long-term feeding with high-concentration feed can lead to subacute rumen acidosis. Therefore, the present study aimed to determine the effect of adding a buffer on subacute rumen acidosis, and the improvement of milk production and milk quality. We also aimed to study the mechanism of promoting mammary gland lactation. A total of 12 healthy mid-lactating goats were randomly divided into two groups, they were high-grain diet group (Control) and buffering agent group. To understand the effects of high-grain diets with buffers on amino acids in jugular blood and the effects of amino acids on milk protein synthesis, Milk-Testing™ Milkoscan 4000, commercial kits, and high-performance liquid chromatography (HPLC) measurements were integrated with the milk protein rate, the amino acid concentration in jugular venous blood samples, quantitative real-time PCR, comparative proteomics, and western blotting to study differentially expressed proteins and amino acids in mammary gland tissues of goats fed high-grain diets. Feeding lactating goats with buffering agent increased the percentage of milk protein in milk, significantly increased the amino acid content of jugular blood (*p* < 0.05), and increase the amino acid transporter levels in the mammary gland. Compared with the high-grain group, 2-dimensional electrophoresis technology, matrix-assisted laser desorption/ionization-time of flight/time of flight proteomics analyzer, and western blot analysis further verified that the expression levels of beta casein (CSN2) and lactoferrin (LF) proteins in the mammary glands of lactating goats were higher when fed a high-grain diets and buffers. The mechanism of increased milk protein synthesis was demonstrated to be related to the activation of mammalian target of rapamycin (mTOR) pathway signals.

## Introduction

In China, there is a lack of high-quality roughage sources; therefore, farmers usually feed their animals with high-concentration feed to obtain high yields and economic benefits. However, long-term feeding of high-concentration feed to ruminants decreases the pH value of ruminants, resulting in abnormal metabolism in the body, which can easily lead to subacute ruminal acidosis (SARA) (1, 2) and will eventually reduce production capacity and milk quality.

A buffer is a chemical substance that can offset and reduce the influence of external strong acid or base on the pH value of a solution, thereby stabilizing the pH value of the solution. In recent years, buffers have been used commonly to prevent acidosis in ruminants and improve the production performance of ruminants in animal husbandry. Experiments have shown that adding 1.5% sodium bicarbonate and 0.8% magnesium oxide to the diets of early and mid-term lactating dairy cows caused milk production and milk fat levels to increase significantly (3).

Kurokawa et al. added 80 g of sodium bicarbonate every day when feeding lactating dairy cows with salt, which was <20% of the standard, and compared with the control group, the standard milk yield increased by 5.1% and the butterfat increased by 0.15% (4). Therefore, in the present study, to maintain the ruminal pH in lactating dairy goats, a buffering agent was added to the high-grain diet.

Milk contains a lot of protein, and protein is one of the most basic components of life. High protein intake can promote the growth and development of the body, can also enhance the body's resistance and reduce the risk of cardiovascular disease (5, 6). Milk protein is one of the determinants of milk quality, and milk protein is mainly synthesized in the mammary glands of lactating ruminants from circulating plasma amino acids (7). Previous studies have shown that amino acids can be taken into the mammary gland by the mammary epithelial cells through transport carriers (8–10). Therefore, it is necessary to understand whether feeding a high-grain diet with buffering agent promotes amino acids flow into the mammary gland through transport carriers amino acids, and to study the signal mechanism of milk protein synthesis.

## Materials and Methods

### Ethics Approval and Consent to Participate

All animal procedures were approved by the Institutional Animal Care and Use Committee of Nanjing Agricultural University. The protocols were reviewed and approved, and the project number 2011CB100802 was assigned. The slaughter and sampling procedures strictly followed the “*Guidelines on Ethical Treatment of Experimental Animals*” (2006) no. 398 established by the Ministry of Science and Technology, China and the “*Regulation regarding the Management and Treatment of Experimental Animals*” (2008) no. 45 set by the Jiangsu Provincial People's Government.

### Experimental Animals

A total of 12 healthy multiparous mid-lactating goats (body weight, 38 ± 8 kg, mean ± SEM, 3–5 weeks post-partum) aged 2–3 years were used in the experiments. They were housed in individual stalls in a standard animal feeding house at Nanjing Agricultural University (Nanjing, China). The goats were randomly divided into two groups, the two groups were respectively high-grain diet group (Control, HG, concentrate: forage = 60:40) and buffering agent group (BG, concentrate: forage = 60:40 with 10 g C_4_H_7_NaO_2_ and 10 g NaHCO_3_ per 1,000 g feed). Dietary C_4_H_7_NaO_2_ and NaHCO_3_ were obtained from Nanjing Jian cheng Bioengineering Institute, China. The ingredients and nutritional composition of the diets are presented in Table 1. During the experimental period of 20 weeks, we fed the goats and obtained their milk from twice daily at 8.00 and 18.00 h. The goats had free access to fresh water, and the feed amount met or exceeded the animal's nutritional requirements. The Institutional Animal Care and Use Committee of Nanjing Agricultural University (Nanjing, People's Republic of China) approved all of the procedures (surgical procedures and care of goats).

**Table 1:** Ingredients and nutritional composition of the diets.

**Concentrate: Forage ratio 60:40**			
**Ingredient (%)**		**Nutrient levels**^**b**^	
Leymus chinensis	27.00	Net energy/(MJ.kg^−1^)	6.71
Alfalfa silage	13.00	Crude protein/%	16.92
Corn	23.24	Neutral detergent fiber/%	31.45
Wheat bran	20.77	Acid detergent fiber/%	17.56
Soybean meal	13.67	Calcium/%	0.89
Limestone	1.42	Phosphorus/%	0.46
NaCl	0.30		
Premix^a^	0.60		
Total	100.00		

a *Provided per kg of diet: VA 6,000 IU/kg, VD 2,500 IU/kg, VE 80 mg/kg, Cu 6.25 mg/kg, Fe 62.5 mg/kg, Zn 62.5 mg/kg, Mn 50 mg/kg, I 0.125 mg/kg, Co 0.125 mg/kg*.

b *Nutrient levels were according to National Research Council (NRC, 2001)*.

### Milk Protein Production Analysis

Samples of the collected milk were analyzed using an Integrated Milk-Testing™ Milkoscan 4000 instrument (Foss Electric, Hillerod, Denmark) at the Animal Experiment Center of College of Animal Science and Technology at the Nanjing Agricultural University.

### Analysis of Total Amino Acids

At the 20th week, blood samples were collected from the jugular blood in 10 mL vacuum tubes containing sodium heparin. Blood was centrifuged at 3,000 × *g* for 15 min to separate the plasma. The total amino acid concentrations were determined using a Total Amino Acid assay kit (catalog no. A026, Jiancheng, Nanjing, China). The procedures were performed according to the manufacturer's instructions.

### Analyses of the Amino Acid Profile Using HPLC

Free amino acids in the jugular blood samples were determined by high performance liquid chromatography (HPLC), as described previously by Shen et al. (11). The HPLC system consisted of: An Agilent 1100 high-performance liquid chromatograph system (Agilent Technologies, Waldbronn, Germany); a scanning fluorescence detector (excitation 340 nm, emission 450 nm); a chromatographic column (XTerra®MS C18, 4.6 × 250 mm, 5 μm), which was purchased from Waters (Milford, MA, USA). Twenty amino acids standards (Sigma Aldrich chemical company, St. Louis, MO, USA) were purchased from Sigma, and the purity of these amino acids were >98%. The gradient elution program of RP-HPLC are presented in Table 2. The three-dimensional flow phase (solution A, methanol; solution B, acetonitrile; solution C, 10 mmol/L phosphate buffer containing 0.3% tetrahydrofuran) was adopted. The oven temperature was 40°C, and the injection volume was 20 μL. The plasma samples were mixed with acetonitrile at 1:2(v/v) and then placed at 4°C for 30 min, before being centrifuged at 12,000 g for 30 min, and the supernatants were collected for amino acid analysis. The HPLC analysis was performed after automatic pre-column derivatization with O-phthaldialdehyde (OPA) (12).

**Table 2:** Gradient elution program of RP-HPLC.

**Time (min)**	**Solution A (%)**	**Solution B (%)**	**Solution C (%)**	**Flow rate (mL/min)**
0	85	6	9	1
10	80	8	12	1
25	70	15	15	1
40	45	25	30	1
50	45	25	30	1

### Quantitative Real-Time Reverse Transcription PCR (qRT-PCR)

After 20 weeks, goats were slaughtered after overnight fasting. All goats were killed by neck vein injections of xylazine (0.5 mg (kg body weight)^−1^; Xylosol; Ogris Pharme, Wels, Austria) and pentobarbital (50 mg (kg body weight)^−1^; Release; WDT, Garbsen, Germany). After slaughter, mammary tissue was collected and washed twice with cold physiological saline (0.9% NaCl) to remove blood and other contaminants. Mammary tissue samples were used for RNA and protein extraction. Total RNA was extracted from each mammary tissue sample using the TRIzol reagent (Invitrogen, Waltham, MA, USA) according to the manufacturer's specifications and then reverse-transcribed into cDNA using M-MLV (H-) Reverse Transcriptase (Vazyme, Nanjing, China). All PCR primers were synthesized by Generay Company (Shanghai, China), and the primer sequences are listed in Table 3. qPCR was performed using an AceQ qPCR SYBR Green Master Mix kit (Vazyme) and an MyiQ2 Real-time PCR system (Bio-Rad, Hercules, CA, USA) with the following cycling conditions: 95°C for 2 min, followed by 40 cycles of 95°C for 15 s and 60°C for 30 s. *GAPDH* (encoding glyceraldehyde-3-phosphate dehydrogenase) served as a reference for normalization.

**Table 3:** Primer sequences used for qRT-PCR analysis of target genes in lactating goats.

**Target genes**	**Primer sequences (5^′^→**3^**′**^**)**	**Products/bp**
SLC1A3	CATCGTGCTGACATCTGTGG/ CCATTTCGACATCCCGGTTC	173
SLC1A5	CATCAACGACTCTGTTGTAGACC/ CGCTGGATACAGGATTGCGG	182
SLC7A5	GAGCGACCCATCAAGGTTCA/ ACCGTCGTGGAAAAGATGCT	206
SLC7A6	AGCAGTGTGGGAAGTAAGGC/ CTGACGCCATTCAGCAGAGA	207
SLC38A2	AGTTCAGTTGGTGGCGTCAT/ CGGTCATCACCACTATGCCA	243
GAPDH	GGGTCATCATCTCTGCACCT/ GGTCATAAGTCCCTCCACGA	180

### Protein Extraction From Mammary Tissues

Protein extraction from mammary tissues was performed according the method described by Duanmu et al. (13). The mammary tissue separately samples in BG and HG are mixed in equal amounts and washed three times with ice-cold saline containing 1 mM phenylmethylsulfonyl fluoride (PMSF), and then homogenized in ice-cold lysis buffer (2 M thiourea, 7 M urea, 50 mM dithiothreitol (DTT), 2% (w/v) 3-[(3-cholamidopropyl) dimethylammonio]-1-propanesulfonate, 0.5% (v/v) Bio-Lyte Ampholyte and 1 mM PMSF) by 1:5 (w/v). The homogenates were kept at room temperature for 30 min, followed by centrifugation at 15,000 × *g* for 30 min at 4°C. The HG samples were treated in the same way. The samples were stored at −80°C until analysis. The protein concentration of the supernatant was determined using an RC DCTM kit (Bio-Rad).

### Two-Dimensional Gel Electrophoresis (2-DE)

The 2-DE method was carried out according to our previously published method (14). The first dimension used was isoelectric focusing (IEF). The extracted protein (1,000 mg) was loaded onto the 17 cm immobilized pH gradient (IPG) gel strips (non-linear, pH 3.0–10.0, Bio-Rad) according to Chen et al. (15) using passive rehydration (13 h with 50 V). IEF was performed with a voltage gradient of 250 V for 1 h, 500 V for 1 h, 2,000 V for 1 h, and 8,000 V for 3 h, followed by holding at 8,000 V until a total of at least 60,000 V-h was reached. Then, the IPG strips were equilibrated by serial incubation for 15 min in equilibration buffer (6 M urea, 30% (v/v) glycerol, 2% (w/v) SDS, 50 mM Tris-HCl (pH 8.8) and 1% (w/v) DTT) and in equilibration buffer containing 2.5% (w/v) iodoacetamide instead of 1% DTT. Equilibrated IPG strips were transferred onto the 12.5% SDS-PAGE for the second dimension (16). Gels were fixed in 12% trichloroacetic acid for 2 h, then stained with 0.08% (w/v) Coomassie Brilliant Blue G250 staining solution for 20 h. The excess dye was removed with MilliQ water, and the gel was scanned using a Molecular Imager (Versa Doc3000, Bio-Rad). Standardization, background elimination, spot detection, gel matching, and interclass analysis were performed as previously described using the PDQuest 8.0 software (Bio-Rad) (17). Three replicates were performed per sample. Protein spots were considered to be differentially expressed only if they showed 1.5-fold change in intensity, and satisfied the non-parametric Wilcoxon test (*p* < 0.05). Only the spots with the same changing trend in all three gels were considered for further analysis.

### Trypsin Digestion and Ms Analysis

Selected gel spots were manually excised and washed twice with MilliQ water. Trypsin digestion of the excised spots was performed as described by Ura et al. (18). The digested proteins were air-dried and analyzed using a 4800 matrix-assisted laser desorption/ionization-time of flight/time of flight (MALDI-TOF/TOF) proteomics analyzer (Applied Biosystems, Foster City, CA, USA). A protein spot digested with trypsin was used to calibrate the mass spectrometer. A mass range of 800–3,500 Da was used. A combined search (mass spectroscopy (MS) plus MS/MS) was performed using GPS Explorer TM software v3.6 (Applied Biosystems) and the MASCOT search engine (Matrix Science Ltd., London, UK). Proteins were considered as positive hits if at least two independent peptides were identified with medium (95%) or high (99%) confidence.

### Western Blotting Analysis

Mixing four different parts of the mammary tissues of six lactating goats each group, and they were mixed in the same amount. Finally, each group got four mixed samples. Protein was extracted from the mixed samples using lysis buffer (Cell Signaling) plus PMSF (1 mM) and total protein was quantified using the bicinchoninic acid (BCA) assay (Pierce, Rockford, IL, USA). We isolated 30 μg of protein from each sample, which was subjected to electrophoresis on SDS-PAGE. The separated proteins were transferred onto nitrocellulose membranes (Bio Trace, Pall Co., Westborough, MA, USA). The blots were incubated with the following primary antibodies overnight at 4°C with a dilution of 1:1,000 in block: Rabbit (rb)-anti-Mammalian target of rapamycin (rb-anti-mTOR, #2983S), rb-anti-phosphorylated-mTOR (rb-anti-p-mTOR, #5536S), rb-anti-P70 ribosomal protein S6 kinase (rb-anti-P70S6K, #9202S), rb-anti-phosphorylated-P70S6K (rb-anti-p-P70S6K, #5536S), rb-anti-eukaryotic translation initiation factor 4E (rb-anti-eIF4E, #9742S), rb-anti-phosphorylated-eIF4E (rb-anti-p-eIF4E, #9741S), rb-anti-eukaryotic elongation factor-2 kinase (rb-anti-eEF2K, #3692S), rb-anti-phosphorylated-eukaryotic elongation factor-2 kinase (rb-anti-p-eEF2K, #3691S), and rb-anti-eukaryotic elongation factor-2 kinase (rb-anti-eEF2, #2332S) (all from Cell Signaling Technology, Danvers, MA, USA). The blots were incubated with primary antibody overnight at 4°C at a dilution of 1:1,000 in block: rb-anti-beta-casein protein (rb-anti-CSN2, #A12749S, ABclonal Technology, Wuhan, China). The blots were incubated with primary antibody overnight at 4°C at a dilution of 1:400 in block: rb-anti-lactoferrin (rb-anti-LF, produced in our laboratory) (19). A rb-anti-GAPDH primary antibody (a531, Bioworld, Nanjing, China, 1:10,000) was also incubated with the blots to provide a reference for normalization. After washing the membranes, they were incubated with horseradish peroxidase-conjugated secondary antibody for 2 h at room temperature. Finally, the blots were washed and the signals were detected using enhanced chemiluminescence (ECL) with the LumiGlo substrate (Super Signal West Pico Trial Kit, Pierce). The ECL signals were recorded using an imaging system (Bio-Rad) and analyzed using Quantity One software (Bio-Rad). The levels phosphorylated mTOR, P70S6K, eIF4E, and eEF2K were determined by the ratio of p- mTOR to total mTOR, p-P70S6K to total P70S6K, p-eIF4E to total eIF4E, and p-eEF2K to total eEF2K, respectively. The expression levels of eEF2, CSN2, and LF were determined by the ratio of eEF2 to GAPDH, the ratio of CSN2 to GAPDH, and the ratio of LF to GAPDH.

### Statistical Analyses

**Figure 1:**
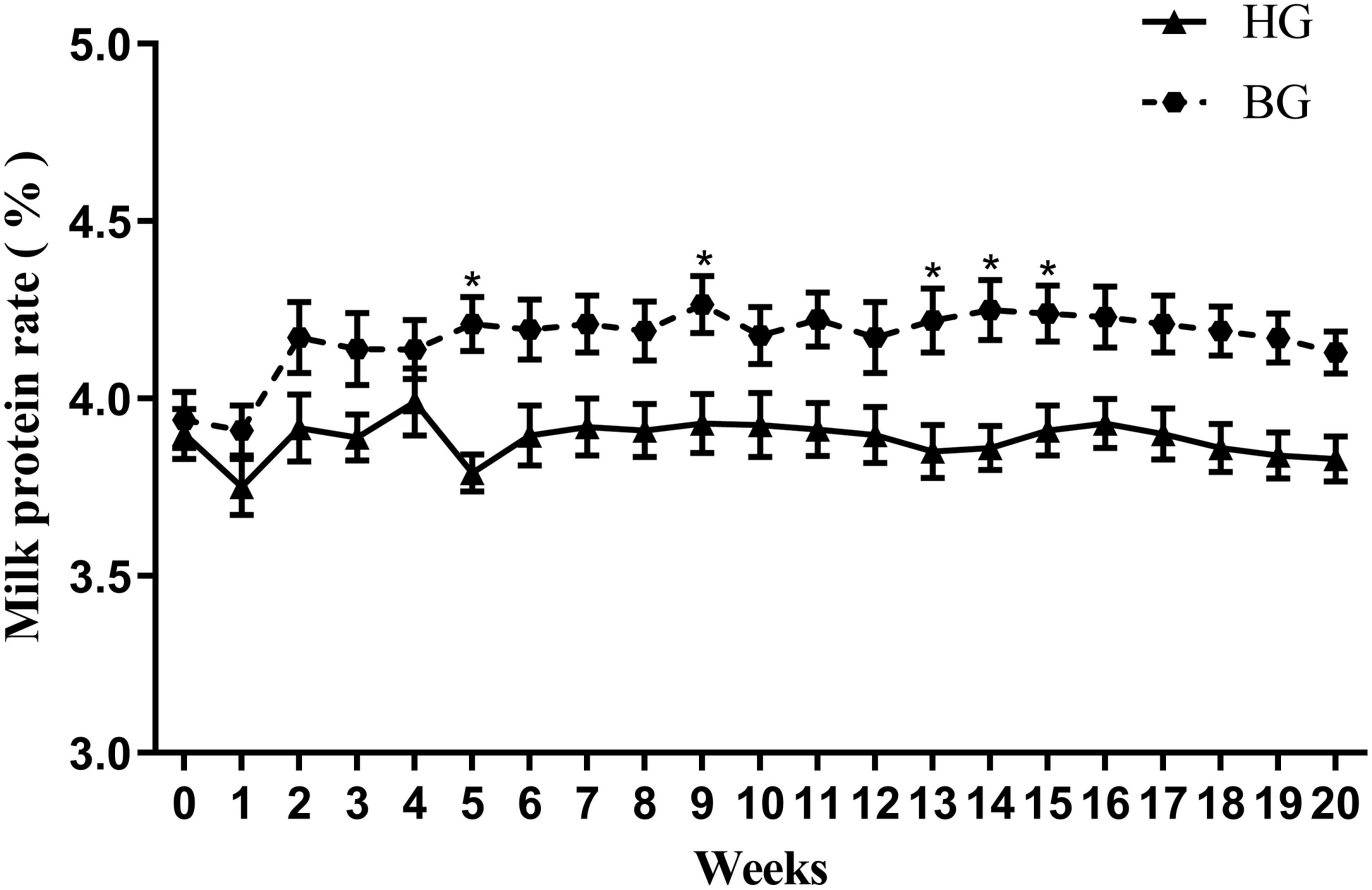
Effect of buffering agents on milk protein rate of lactating goats. Values are mean ± SEM, *n* = 6/group. *p < 0.05, compared with high grain group.

**Figure 2:**
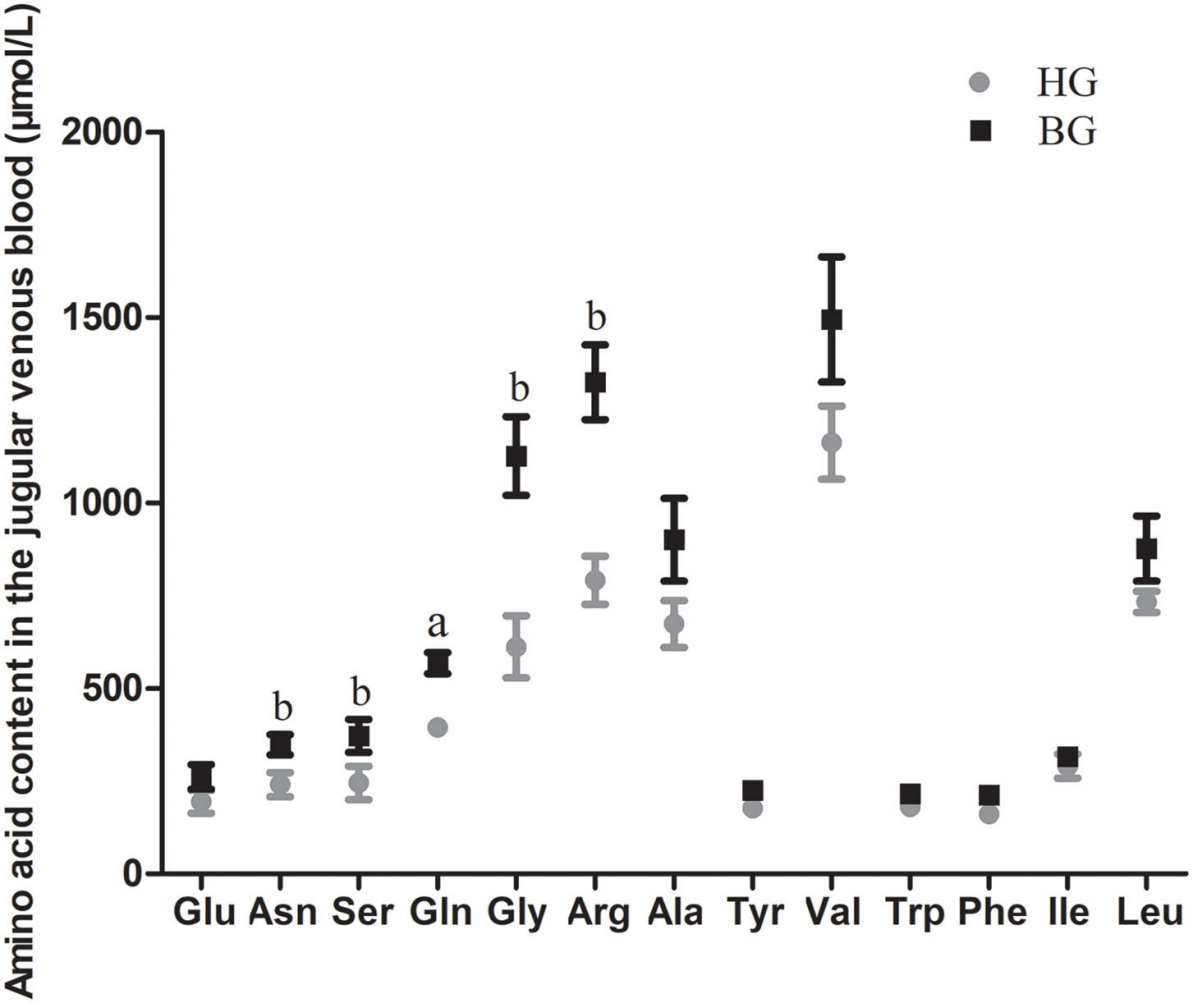
Impact of high-grain diet with buffering agent on amino acid content in plasma of lactating goats. Values are mean ± SEM, *n* = 6/group. ^b^*p* < 0.05 and ^a^*p* < 0.01, compared with high grain group.

**Figure 3:**
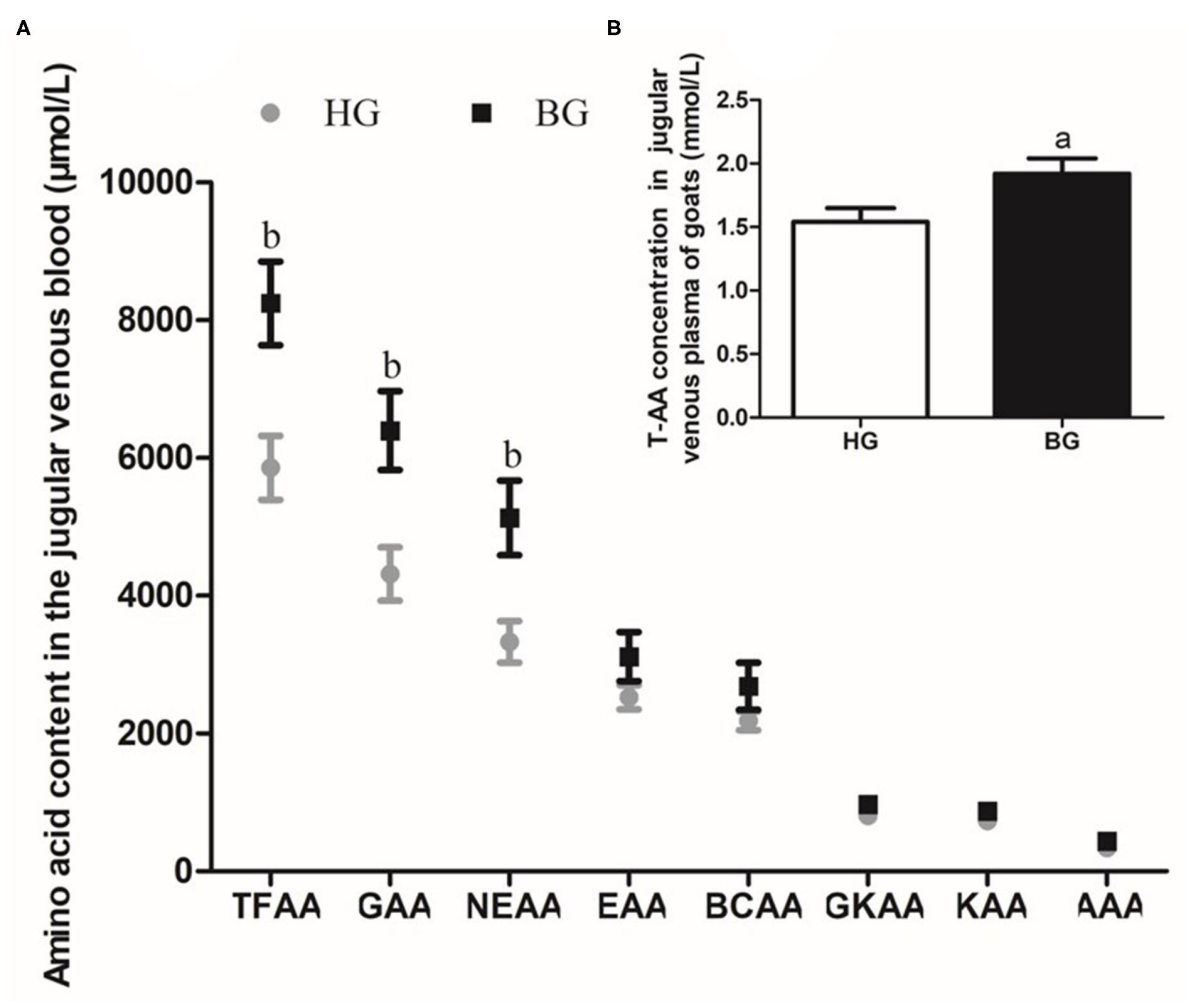
Different types of amino acids content in plasma of lactating goats. **(A)** Impact of high-grain diet with buffering agent on different types of amino acids content in plasma of lactating goats by HPLC. **(B)** Impact of high-grain diet with buffering agent on total amino acids content in plasma of lactating goats by Total Amino Acid assay kit. Values are mean ± SEM, *n* = 6/group. ^b^*p* < 0.05 and ^a^*p* < 0.01, compared with high grain group. TFAA, total free amino acid; GAA, glycogenic amino acid; NEAA, non-essential amino acid; EAA, essential amino acid; BCAA, branched-chain amino acid; GKAA, glucogenic and ketogenic amino acid; KAA, ketogenic amino acid; AAA, aromatic amino acid.

**Figure 4:**
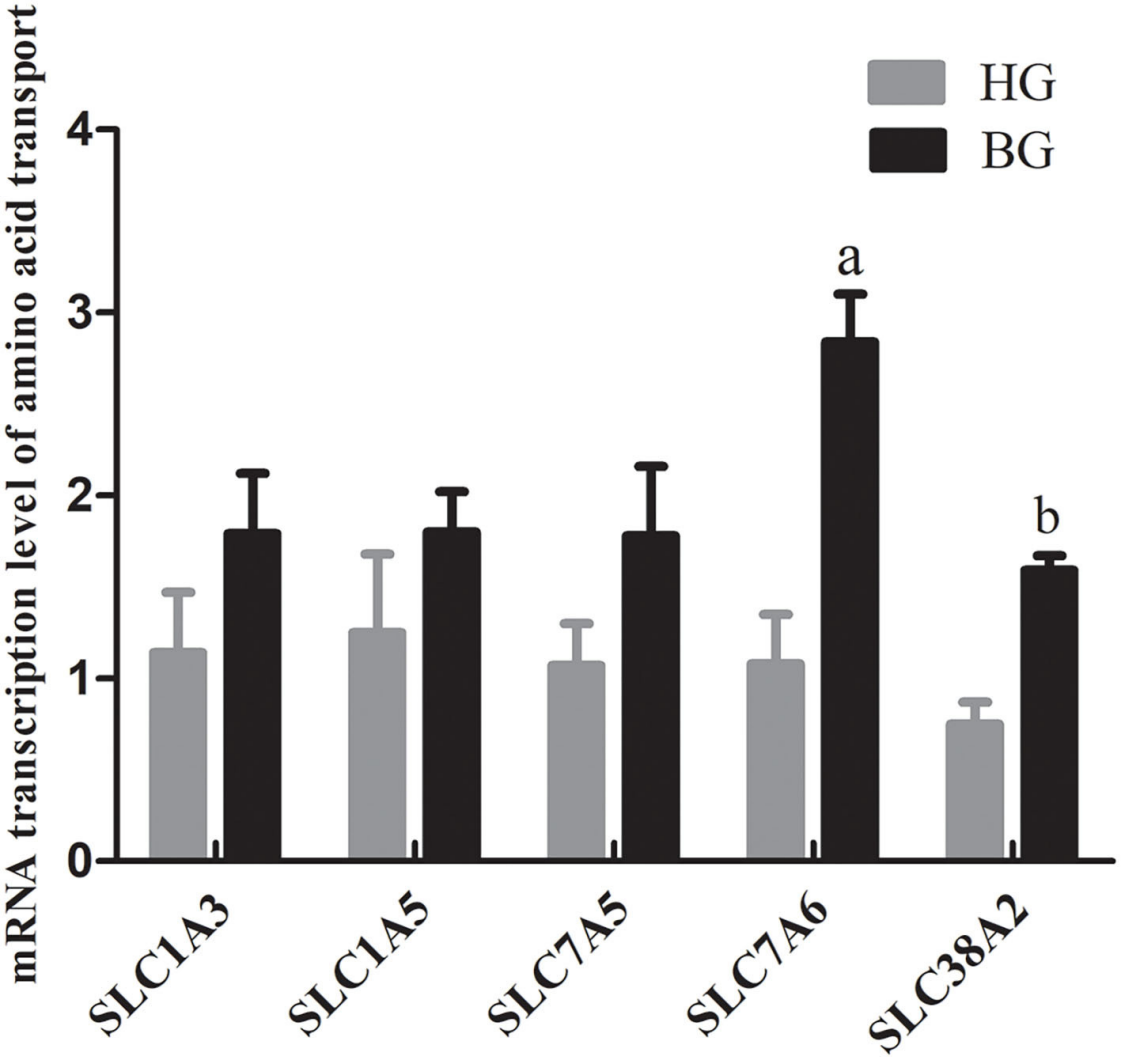
mRNA expressions of amino acid transport SLC1A3, SLC1A5, SLC7A5, SLC7A6, and SLC38A2. Each sample was first normalized against its GAPDH transcript level, and then normalized to the HG. In order to calculate differences in the expression level of each target gene, the 2^−Δ*ΔCt*^ method for relative quantification was used, according to the manufacturer's manual. Values are mean ± SEM. *n* = 6/group. ^b^*p* < 0.05 and ^a^*p* < 0.01, compared with high grain group.

**Figure 5:**
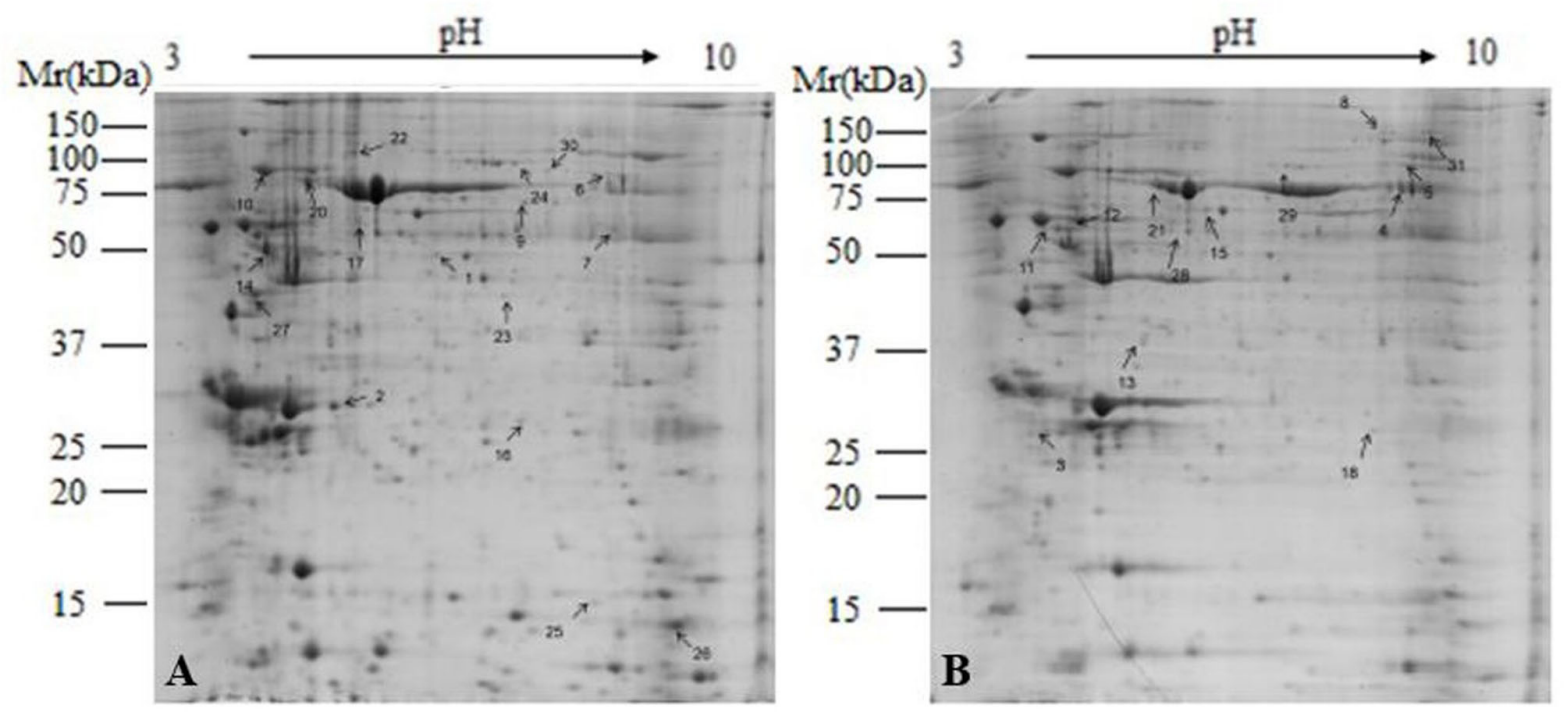
Representative 2-DE images of proteins extracted from lactating goat mammary. **(A)** High-grain group; **(B)** Buffering agent group. Equal amounts of protein (1,000 mg) were loaded onto the 17 cm IPG gel strip (non-linear, pH 3.0–10.0), and separated on 17-cm IPG strips (pH 3.0–10.0), followed by electrophoresis on 12.5% SDS-PAGE gels for second dimension electrophoresis. Black arrows indicate differential protein spots (≥1.5-fold).

**Figure 6:**
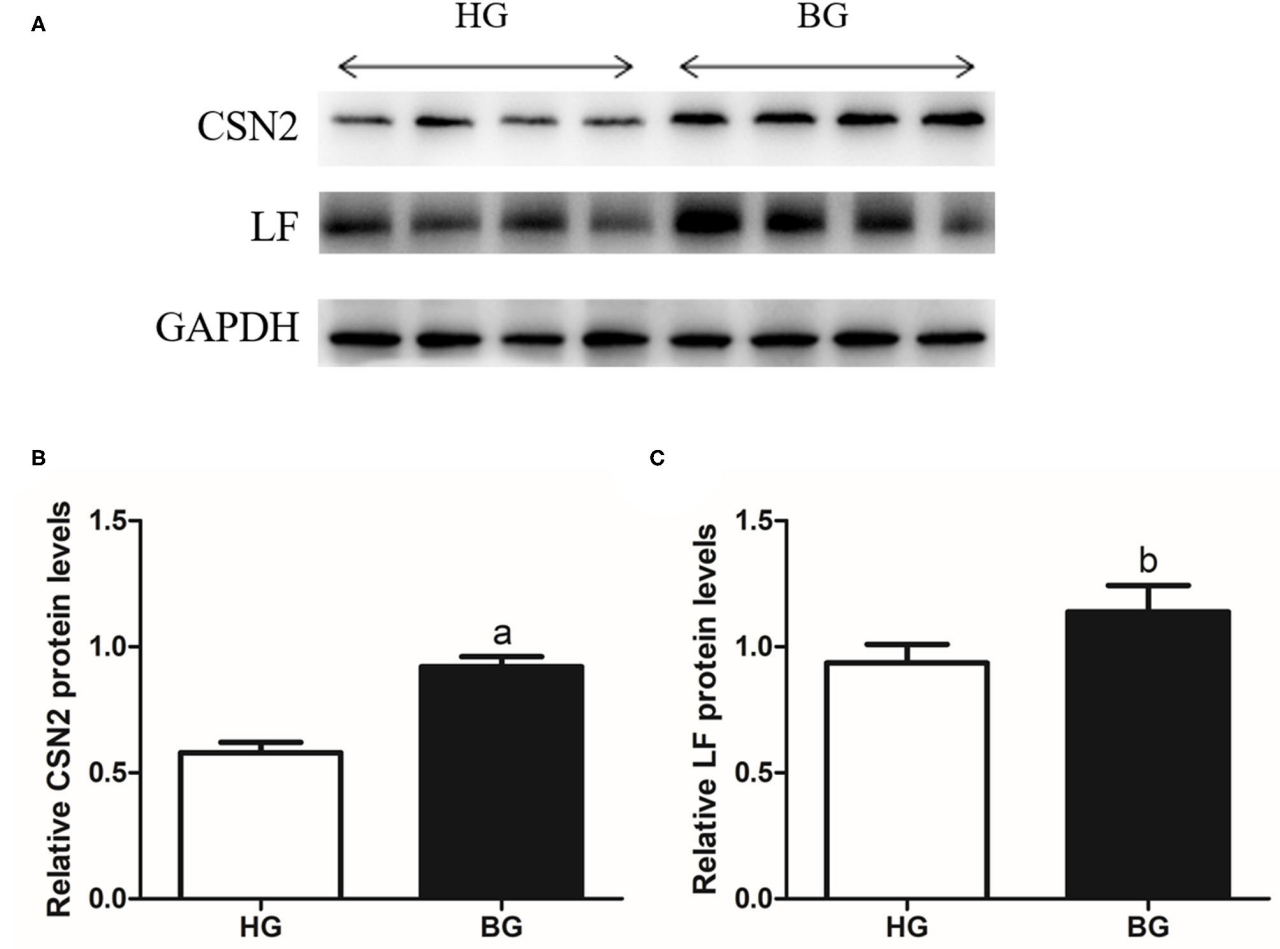
Western blot analysis of the CSN2 and LF. **(A)** Indicated protein levels were measured by western blotting analysis. **(B,C)** Relative protein levels of CSN2 **(B)** and LF **(C)** from the western blots were quantified by gray scale scan. Values are mean ± SEM. *n* = 4/group. ^b^*p* < 0.05 and ^a^*p* < 0.01, compared with high grain group.

**Figure 7:**
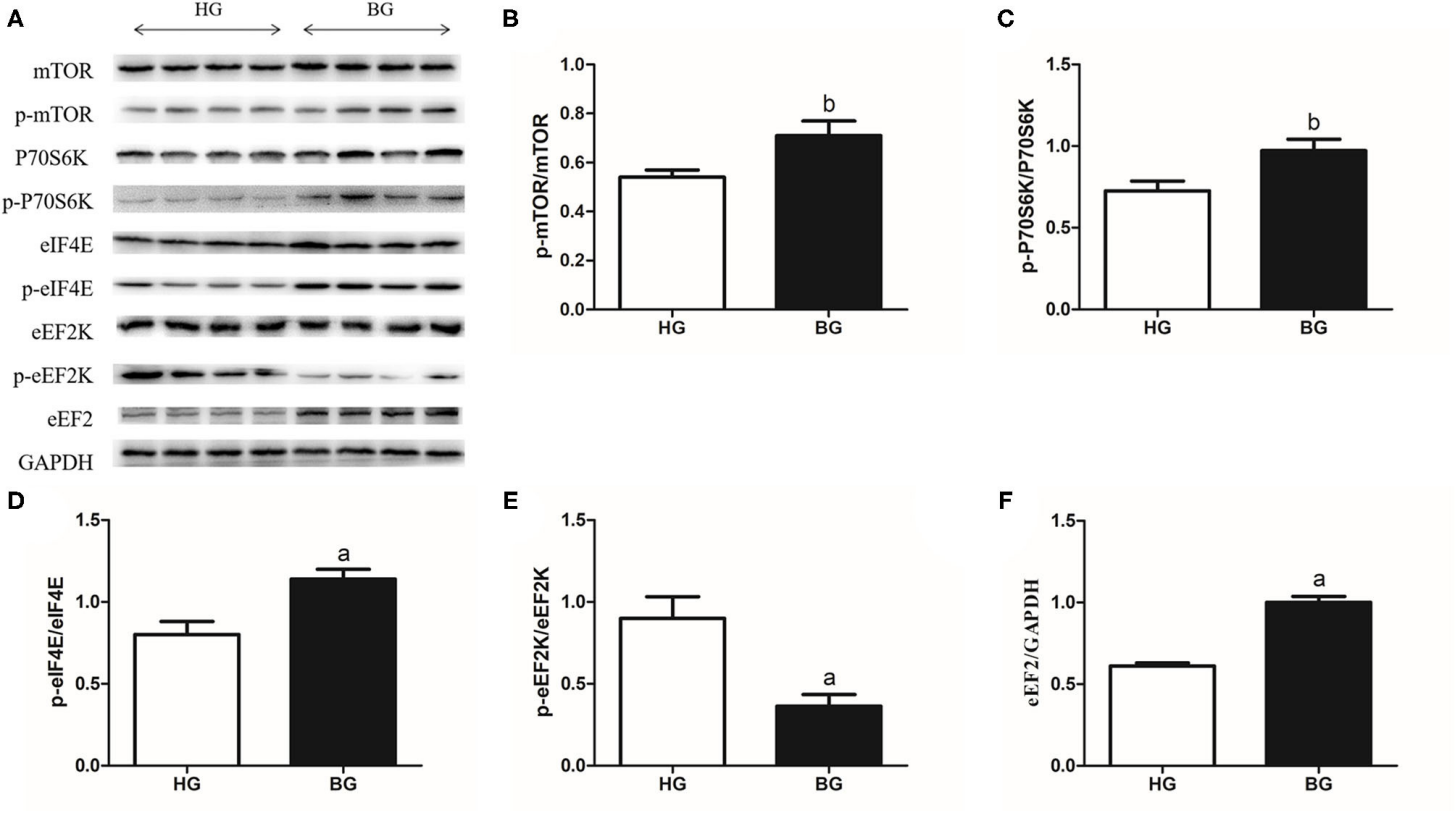
Milk protein synthesis mTOR related signaling pathway protein expressions. **(A)** mTOR related signaling pathway protein expression levels were measured by western blotting analysis. **(B)** The ratios of phosphorylated to total mTOR were quantified by gray scale scan. **(C)** The ratios of phosphorylated P70S6K to total P70S6K were quantified by gray scale scan. **(D)** The ratios of phosphorylated eIF4E to total eIF4E were quantified by gray scale scan. **(E)** The ratios of phosphorylated eEF2K to total eEF2K were quantified by gray scale scan. **(F)** Relative protein levels of eEF2 from the western blots were quantified by gray scale scan. Values are mean ± SEM. *n* = 4/group. ^b^*p* < 0.05 and ^a^*p* < 0.01, compared with high grain group.

Data are presented as the means ± SEM. Data were tested for normal distribution, and statistical significance was assessed by the independent sample *t*-test using SPSS version 17.0 for Windows (SPSS Inc., Chicago, IL, USA). Data were considered statistically significant if *P* < 0.05. The numbers of replicates used for statistics are noted in the Figures 1–**7**.

## Results

### Effect of Buffering Agents on Milk Protein Proportion

As shown in Figure 1, the percentage of milk protein in the weekly milk production after buffering was different from that of the high grain group. The milk protein percentage in BG was higher than that in HG at 20 weeks, there was a significant difference at week 9, 13, 14, and 15 (*P* < 0.05).

### Amino Acid Content in Jugular Venous Plasma

As shown in Figure 2, the concentrations of 14 amino acids were higher in the buffering agent group than in the high grain group. The content of Gln was very significantly higher in BG than in HG (*P* < 0.01). The amino acid concentrations of Asn, Ser, Gly, and Arg were significantly higher in BG than in HG (*P* < 0.05).

### Different Types of Amino Acids in Jugular Venous Plasma

As shown in Figure 3A, the total amount of free amino acids (TFAAs), glycogenic amino acids (GAAs), and non-essential amino acids (NEAAs) were significantly higher in BG than in HG (*P* < 0.05) (Figure 3A). In addition, the total AA content was significantly higher in BG compared with that in HG (*P* < 0.01) (Figure 3B).

### mRNA Transcription Level of Amino Acid Transporter Proteins

The excitatory amino acid transporter 1 (EAAT1, encoded by *SLC1A3*), alanine-serine-cysteine transporter 2 (ASCT2, encoded by *SLC1A5*), L-type amino acid transporter 1 (LAT1, encoded by *SLC7A5*), sodium-independent neutral and basic amino acid transporter (y^+^LAT2, encoded by *SLC7A6*), and sodium-coupled neutral amino acid transporter 2 (SNAT2, encoded by *SLC38A2*) are transporters of amino acids, which are required to maintain cell growth and protein synthesis in ruminants (20–22). However, the function of these amino acid transporters in regulating milk protein synthesis in the mammary glands of lactating goats is unknown. Therefore, the mRNA expression levels of these genes encoding amino acid transporters in the mammary gland were detected using qRT-PCR. The mRNA expression of *SLC38A2* was significantly increased (*P* < 0.05), and the mRNA expression of *SLC7A6* was very significantly increased (*P* < 0.01) in BG goats compared with those in HG goats (Figure 4).

### Global Identification of Differentially Abundant Proteins in the Mammary Gland

To understand the effect on mammary gland metabolism of adding buffers, a comparative proteomic analysis was carried out on the mammary gland tissues of HG and BG in lactating Saanen goats. As shown in Figure 5, an average of 1,200 spots were detected on gels for both types of proteomes. A total of 32 differential protein spots (*P* < 0.05; in terms of expression, all 32 with a fold change ≥1.5-fold) were successfully identified in BG vs. HG using 2-DE technique and the MALDI-TOF/TOF proteomics analyzer. Among them, 15 proteins showed increased expression and 17 proteins showed decreased expression in BG vs. HG.

We observed that the main differentially abundant proteins were related to amino acid metabolism, glucose metabolism, lipid metabolism, oxidative stress, mitochondrial function, cytoskeleton structure, and immune proteins (Table 4).

**Table 4:** Differential expression protein spots by MAIDI-TOF-TOF.

**Spot no**.	**Identified protein name**	**Gene symbol**	**Accession no**.	**Mr (kDa)**	**pI**	**Protein expression**	**1.5-fold change**
**Amino acid metabolism**						
1	Ornithine aminotransferase	OAT	gi|803207295	37.8	8.37	Down	0.65
2	Beta casein	CSN2	gi|1211	24.9	5.26	Up	2.73
3	Peptidylproly l isomerase A	PPIA	gi|410066839	18.0	8.34	Down	0.66
4	Lactoferrin	LF	gi|56544486	79.2	8.4	Up	2.27
**Glucose Metabolism**						
5	Pyruvate kinase PKM isoform X7	PKM	gi|426232638	58.5	7.60	Down	0.23
6	UTP–glucose-1-phosphate uridylyltransferase isoform X1	UGP2	gi|426223460	57.0	7.68	Up	2.13
7	Transketolase	TKT	gi|426249391	68.5	7.2	Up	2.52
8	Aconitate hydratase, mitochondrial isoform X1	ACO2	gi|803058827	95.9	6.78	Down	0.5
9	Aldehyde dehydrogenase, mitochondrial	ALDH2	gi|426247368	57.1	7.55	Down	0.52
10	Glucose-regulated protein	HSPA5	gi|426223038	72.5	5.07	Up	1.66
**Cytoskeletal structure**						
11	Tubulin	Tubulin	gi|257097261	50.2	4.78	Up	1.66
12	Vimentin	VIM	gi|145226795	53.7	5.02	Up	1.56
13	F-actin-capping protein subunit beta isoform X1	CAPZB	gi|426222052	34.0	5.82	Down	0.49
14	Protein disulfide-isomerase A6	PDIA6	gi|803229999	48.5	4.95	Up	2.1
**Lipid metabolism**						
15	Glycerol kinase isoform X5	GK	gi|426256814	61.4	5.60	Down	0.41
16	Acid synthase isoform X2	FASN	gi|803208475	27.6	5.93	Up	6
**Oxidative stress**						
17	Cytochrome b-c1 complex subunit 1	UQCRC1	gi|803186314	51.9	6.03	Down	0.42
18	Glutathione S-transferase P	GSTP1	gi|803225108	23.8	6.89	Up	1.96
**Mitochondrial function**						
19	ATP synthase subunit beta, mitochondrial	ATP5B	gi|426224929	56.2	5.15	Up	1.66
20	NADH-ubiquinone oxidoreductase 75 kDa subunit	NDUFS1	gi|426221412	80.4	5.90	Down	0.46
**Immune protein**						
21	Pre-pro serum albumin	ALB	gi|1387	71.1	5.80	Down	0.18
22	Polymeric immunoglobulin receptor	PIGR	gi|426239425	83.7	5.79	Down	0.17
23	Annexin A1	ANXA1	gi|426220300	39.0	6.17	Down	0.47
24	Serotransferrin isoform X2	TF	gi|803335974	79.4	6.31	Up	2.38
25	Beta globin chain	HBB	gi|86129745	16.0	6.75	Up	1.89
26	Alpha globin chain	HBAI	gi|1787	15.3	8.72	Up	1.60
**Other**						
27	Protein FAM186B isoform X1	FAM186B	gi|803043984	111.3	8.87	Down	0.47
28	T-complex protein 1 subunit epsilon	CCT5	gi|426246714	60.03	5.6	Down	0.57
29	Radixin isoform X1	RDX	gi|803154243	68.4	6.03	Down	0.53
30	Lamin isoform X2	LMNA	gi|803255736	74.4	6.76	Down	0.48
31	Elongation factor 2 isoform X2	EEF2	gi|426229147	96.2	6.41	Down	0.36
32	cytochrome b-c1 complex subunit 2	UQCRC2	gi|426254425	48.8	8.89	Up	2.47

Beta casein (CSN2) is the main component of milk protein. Lactoferrin (LF) is a non-heme iron-binding glycoprotein in milk and is a member of the transferrin family. During lactation, LF is expressed and secreted by mammary epithelial cells at the mucosal surface. The protein levels of CSN2 and LF were upregulated in BG compared with those in HG.

### Validation of Differentially Abundant Milk Proteins

To further validate the levels of CSN2 and LF proteins (the main components of milk protein), we performed western blotting analysis (Figure 6). The CSN2 protein level was very significantly higher in BG than in HG (*P* < 0.01) and the LF protein level was significantly higher in BG than in HG (*P* < 0.05).

### The Signaling Pathway for Milk Protein Synthesis

Western blotting was applied to study the mechanism of milk protein synthesis. The results of these protein expression levels were showed in Figure 7A. The results showed that the mTOR phosphorylation ratio and the P70S6K phosphorylation ratio were upregulated significantly in BG compared with that in HG (*P* < 0.05, Figures 7B,C), the eIF4E phosphorylation ratio and the level of eEF2 were upregulated significantly (*P* < 0.01, Figures 7D,F); however, the eEF2K phosphorylation ratio was downregulated (*P* < 0.01, Figure 7E). These results suggested that adding a buffering agent increased the phosphorylation ratios mTOR, P70S6K, and eIF4E, increased the level of eEF2, and decreased the eEF2K phosphorylation ratio.

## Discussion

Milk production mainly depends on fat synthesis, milk protein production, and the proliferation capacity of mammary epithelial cells (23). Milk proteins in the lactating ruminant mammary gland are primarily synthesized from circulating plasma amino acids, present in both free and peptide-bound forms, and are used by tissues both as building blocks for protein synthesis and as signaling molecules to regulate the protein synthetic machinery (24, 25). The mammary gland requires large amounts of amino acids to synthesize milk protein. During lactation, the mammary glands of ruminants must not only acquire amino acids from plasma, but also endogenously synthesize sufficient amounts of amino acids to increase milk protein synthesis (26). Identifying and understanding the changes of amino acids in plasma and amino acids transport into the lactating mammary gland will provide fundamental knowledge to promote the development of nutritional regimes aimed at elevating milk production. In the present study, we found that the concentrations of 14 free amino acids were higher, the amino acid concentrations of TFAAs, GAAs, and NEAAs were significantly higher (*P* < 0.05), the total AA concentration in the plasma of lactating goats was also very significant higher (*P* < 0.01) in BG compared with that in HG. Therefore, compared with long-term feeding of a high-grain diet, the addition of buffers increased the concentration of amino acids in the blood.

Presently, the amino acid transporter system in the mammary gland is not well-understood; however, a study suggested that the mammary gland has a transport system similar to that in other organs (intestine, kidney, placenta) (27). We investigated the main transporters involved in amino acid transport and uptake in lactating goat's mammary gland. We found that the mRNA expression levels of *SLC1A3, SLC1A5, SLC7A5, SLC7A6*, and *SLC38A2* were upregulated in mammary tissue from BG goats compared with those in HG goats. EAAT1, encoded by *SLC1A3*, is an isoform of the ${\text{X}}_{\text{AG}}^{-}$ system and is a Na^+^-dependent transporter with high affinity for Asp and Glu. ASCT2, encoded by *SLC1A5*, is Na^+^-dependent and has affinity for small neutral AAs, such as Ala, Ser, and Cys (28). y^+^LAT2, encoded by *SLC7A6*, is a catalytic light chain and is linked to a heavy subunit (4F2hc) via a disulfide bond to form a heteromeric Na^+^-dependent transporters, y^+^LAT2/4F2hc (29). y^+^LAT2/4F2hc is located in the basolateral cell membrane, where it functions as an obligate asymmetric AA exchanger and has affinity for Lys, Arg, Gln, His, Met, Leu, Ala, and Cys (30). LAT1, encoded by *SLC7A5*, is found in many different types of mammalian cells, and is indispensable as a transporter of essential AAs to maintain cell growth and protein synthesis (31). SNAT2, encoded by *SLC38A2*, is expressed in the mammary gland and plays an important role in the uptake of alanine and glutamine, which are the most abundant amino acids transported into this tissue during lactation. SNAT2 also has affinity for Gly, Pro, Ala, Ser, Cys, Gln, Met, His, and Asn. The expression of *SLC38A2* can be upregulated by amino acids and hormones (prolactin and 17β-estradiol) (32). Taken together with these previous reports, our experimental data suggest that adding a buffering agent could not only promoted increased amino acid concentrations in blood, but also, through increased amino acid transport, allowed more amino acids from the blood to enter the mammary gland for the synthesis of milk protein compared with long-term feeding of high grain diet. Further studies to identify changes in mammary genes involved in amino acid transport and uptake in the mammary gland are required.

Milk protein is mainly composed of casein and whey protein. The protein content in goat milk is about 2.1–3.5%. In goat milk, the ratio of casein to whey is about 75:25. Casein is synthesized by mammary epithelial cells (33). It is a group of phosphoproteins unique to milk and contains a large amount of phosphorus and calcium. The casein fraction consists of α_s1_-, α_s2_-, β-, and κ-casein. It has been reported that β-casein has the highest content among caseins in goat's milk, which is different from the cow's milk (34). The whey fraction consists of mainly β-lactoglobulin (β-lg), α-lactalbumin (α-la), immunoglobulins, lactoferrin (LF), and lysozyme (35–37). To verify that the increase in amino acids entering the mammary gland are utilized by the mammary gland to synthesize more milk proteins, we used 2-DE and MALDI-TOF/TOF proteomics. Compared with HG, we observed upregulated levels of CSN2 and LF in BG. Western blotting verified the increased levels of CSN2 and LF in BG compared with that in HG.

The results show that adding buffers to the high-grain diet can promote the synthesis of casein, which is the main component of milk protein, and lactoferrin, which is a component of whey protein in the mammary glands, thus increasing milk protein production. However, the shortcoming of this experiment is that no further analysis of the milk protein in the collected milk was done, otherwise the results of the 2-DE and western blotting would be more convincing.

The mammalian target of rapamycin (mTOR) is a multiple component of protein synthesis and is the key regulator of milk protein synthesis. Most reports have focused on the role of amino acids and mTOR in protein synthesis (38–40). However, whether the entry of acids into the mammary gland to regulate the mTOR signaling pathway and promote milk protein synthesis is not yet clear. To further explore the mechanism by which adding a buffer regulates the expression of related proteins in milk protein synthesis, we studied the activity of the mTOR signaling pathway. The results indicated that amino acids could activate mTOR signaling, which is consistent with previous reports (41). We determined that the level of mTOR phosphorylation is an indicator of activation of the mTOR pathway, which is increased by increased amino acid entry into the mammary gland. mTORC1 activates P70S6K through phosphorylation, which in turn activates several proteins that help to increase protein synthesis. In addition, activation of P70S6K inhibits eEF2K through phosphorylation, and subsequently stimulates eEF2, thereby activating translocation elongation. The phosphorylation of mTORC1 leads to inhibition of 4E binding protein 1 (4EBP1) through phosphorylation, thereby inhibiting eIF4E. eIF4E activation relies on cap translocation, thereby increasing protein synthesis. Through these different proteins, mTORC1 then stimulates cell growth by increasing cap-dependent translocation, translation elongation, mRNA biogenesis, and ribosomal biogenesis, leading to an increase in overall protein synthesis.

## Conclusions

**Figure 8:**
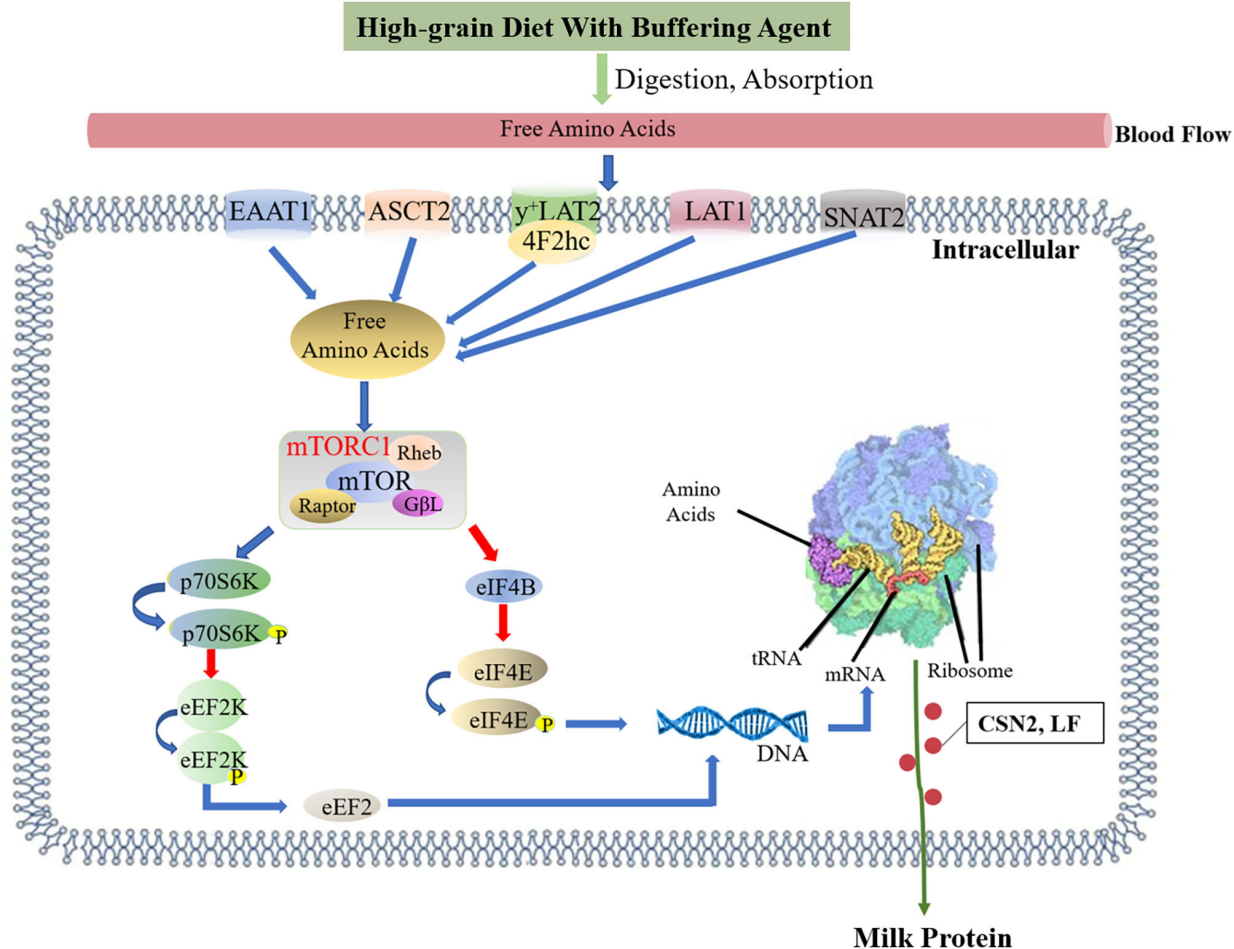
Effects of high-grain diet with buffering agent on the milk protein synthesis. Feeding of high-grain diet with buffering agent promoted the jugular vein blood of amino acids concentration, and the mRNA expressions of amino acid transport SLC1A3, SLC1A5, SLC7A5, SLC7A6, and SLC38A2 in mammary tissues were up-regulated, it showed that more amino acids flowed into the mammary. Amino acids in mammary gland activated mTOR pathway. mTORC1 activates P70S6K via phosphorylation, which in turn activated several proteins that contributed to promote protein synthesis. In addition, activation of P70S6K inhibited eEF2K via phosphorylation, subsequently stimulated eEF2, which activated translocation elongation. When mTORC1 was phosphorylated, it inhibited 4EBP1 through phosphorylation, which inhibits eIF4E. eIF4E activates cap-dependent translocation increasing protein synthesis. The blue arrow represents promotion, and the red arrow represents inhibition.

The results suggested that adding a buffer to high-grain diet could increase the milk protein content of amino acids in mammary gland milk and jugular blood, and increase the mRNA expression levels of amino acid transporters in mammary gland tissue. Adding a buffer upregulated the flow of amino acids into the mammary glands. Proteomic and western blotting analyses further confirmed the increase in CSN2 and LF protein synthesis in mammary gland, which is suggested to be related to the activation of the mTOR signaling pathway (Figure 8).

## Data Availability Statement

The original contributions presented in the study are included in the article/supplementary material, further inquiries can be directed to the corresponding author/s.

## Ethics Statement

The animal study was reviewed and approved by the Institutional Animal Care and Use Committee of Nanjing Agricultural University.

## Author Contributions

MH, HW, SY, and YZ performed the experiments, including the collection of samples, clinical evaluation, sequencing, and data analysis. MH and XN analyzed the data. MH and YZ drafted and revised the manuscript. All authors contributed to conception and design of the project, approved the final version of the manuscript, and agree to be accountable for all aspects of the work in ensuring that questions related to the accuracy or integrity of any part of the work are appropriately investigated and resolved.

## Conflict of Interest

The authors declare that the research was conducted in the absence of any commercial or financial relationships that could be construed as a potential conflict of interest.
